# Caffeine in Aging Brains: Cognitive Enhancement, Neurodegeneration, and Emerging Concerns About Addiction

**DOI:** 10.3390/ijerph22081171

**Published:** 2025-07-24

**Authors:** Manuel Glauco Carbone, Giovanni Pagni, Claudia Tagliarini, Icro Maremmani, Angelo Giovanni Icro Maremmani

**Affiliations:** 1 Division of Psychiatry, Department of Medicine and Surgery, University of Insubria, Viale Luigi Borri 57, 21100 Varese, Italy; manuelglaucocarbone@gmail.com; 2 VP Dole Research Group, G. De Lisio Institute of Behavioural Sciences, Via di Pratale 3, 56121 Pisa, Italy; angelogiovanniicro.maremmani@unicamillus.org; 3 Saint Camillus International University of Health Sciences, Via di Sant’Alessandro 8, 00131 Rome, Italy; 4 Department of Psychiatry, North-Western Tuscany Local Health Unit, Tuscany NHS, Lunigiana Socio-Sanitary Area, Piazza Craxi 22, 54011 Aulla, Italy; giovanni.a.pagni@gmail.com; 5 Psychiatric Diagnosis and Treatment Service (S.P.D.C.), Sant’Elia Hospital, Provincial Health Authority 2, Via Luigi Russo 6, 93100 Caltanissetta, Italy; claudiatagliarini8@gmail.com

**Keywords:** caffeine dependence, neurodegenerative diseases, cognitive impairment, elderly

## Abstract

This narrative review examines the effects of caffeine on brain health in older adults, with particular attention to its potential for dependence—an often-overlooked issue in geriatric care. Caffeine acts on central adenosine, dopamine, and glutamate systems, producing both stimulating and rewarding effects that can foster tolerance and habitual use. Age-related pharmacokinetic and pharmacodynamic changes prolong caffeine’s half-life and increase physiological sensitivity in the elderly. While moderate consumption may enhance alertness, attention, and possibly offer neuroprotective effects—especially in Parkinson’s disease and Lewy body dementia—excessive or prolonged use may lead to anxiety, sleep disturbances, and cognitive or motor impairment. Chronic exposure induces neuroadaptive changes, such as adenosine receptor down-regulation, resulting in tolerance and withdrawal symptoms, including headache, irritability, and fatigue. These symptoms, often mistaken for typical aging complaints, may reflect a substance use disorder yet remain under-recognized due to caffeine’s cultural acceptance. The review explores caffeine’s mixed role in neurological disorders, being beneficial in some and potentially harmful in others, such as restless legs syndrome and frontotemporal dementia. Given the variability in individual responses and the underestimated risk of dependence, personalized caffeine intake guidelines are warranted. Future research should focus on the long-term cognitive effects and the clinical significance of caffeine use disorder in older populations.

## 1. Introduction

The consumption of caffeine among the elderly represents a complex issue that warrants careful and nuanced examination. While it is acknowledged that caffeine intake can confer certain benefits, it is equally important to evaluate potential risks and the heightened sensitivity that this age group may develop. Caffeine is not solely found in coffee; it is also present in tea, chocolate, energy drinks, and even in some pharmaceuticals, which contributes to its widespread exposure across populations [1,2]. For the purposes of clarity and consistency in this review, we will use the term “elderly people” to refer to individuals aged 65 years or older, a definition that aligns with criteria commonly used by the World Health Organization (WHO) and in numerous studies in the field of geriatrics. It is important to acknowledge, however, that a universally accepted definition of “elderly” does not exist, and that the studies included in this review may employ varying age-based inclusion criteria (e.g., 60 years and older). We recognize that this variability could be a limitation when interpreting and comparing research findings.

In older adults, the metabolism of caffeine tends to slow, leading to prolonged biological half-life and amplification of its effects, thus necessitating a cautious approach to consumption [3].

A key factor contributing to this prolonged half-life is the age-related decline in metabolic rate. However, compromised renal function can further impair the clearance of caffeine metabolites, exacerbating this effect in older adults. Supporting the influence of genetic factors and their association with kidney function, recent research [4,5] has confirmed these relationships. Furthermore, a cross-sectional analysis of data from the National Health and Nutrition Examination Survey (NHANES)—a nationally representative survey assessing the health and nutritional status of adults and children in the United States—by Gao et al. (2025) [6] highlighted an inverse association between the consumption of coffee, tea, and caffeine and the presence of CKD, further reinforcing the potential protective role of these dietary habits on renal health.

Moderate caffeine intake has generally been associated with cognitive benefits in elderly individuals, including improvements in short-term memory, attention, and verbal fluency [7,8,9]. Nonetheless, with advancing age, sensitivity to caffeine appears to increase, consequently elevating the risk of adverse effects [10]. These include sleep disturbances, heightened anxiety, gastrointestinal issues, cardiovascular problems, and an increased likelihood of developing osteoporosis [11,12,13,14,15]. Moreover, age-related decline in renal function, coupled with reduced hydration status and potential interactions with polypharmacy, may exacerbate adverse outcomes related to caffeine consumption [16,17,18].

Therefore, a careful assessment of caffeine intake is essential in elderly populations, considering individual health conditions, comorbidities, and medication regimens to balance potential benefits against risks accurately. An often underestimated aspect is the potential for dependence; even in late adulthood, habitual consumers may develop caffeine dependence, with abrupt reduction potentially provoking withdrawal symptoms, such as headaches, fatigue, and irritability [19,20,21].

The prevalence of caffeine consumption among older adults is considerable and warrants attentive awareness from healthcare professionals. Epidemiological studies have demonstrated that a significant proportion of individuals over 65 years consume caffeine regularly [22,23,24]. For instance, a 2018 survey conducted by the National Sleep Foundation reported that approximately 40% of Americans aged between 65 and 79, and about 35% of those aged 80 and above, consume caffeine on a daily basis [25,26,27]. Although research specifically targeting caffeine dependence in older populations remains limited, some evidence suggests that the risk of developing dependence may increase with age, particularly after 60 years [21,28,29,30,31]. This trend could be attributed to factors like decreased metabolic rate and physiological changes in body composition, which may render older adults more susceptible to the stimulating effects of caffeine [32,33].

Caffeine consumption is also widespread within the Italian elderly population, although data indicate that prevalence decreases with age [34]. A survey by the Italian National Institute of Statistics revealed that around 70% of Italians aged between 65 and 74 drink coffee daily [35]. Notably, compared to US figures, Italians tend to consume higher quantities of caffeine overall [36,37]. Nonetheless, it appears that elderly Italians may have a lower propensity to develop caffeine dependence compared to their American counterparts, possibly due to cultural differences in consumption patterns and genetic factors influencing caffeine metabolism [21,38,39,40]. It is important to acknowledge that environmental factors, particularly climate and humidity, can influence renal capacity and hydration, potentially impacting caffeine metabolism [41,42]. While these factors may contribute to population-level differences in caffeine sensitivity, definitive evidence remains limited.

To further elucidate individual variability, we considered research on genetic variations influencing caffeine metabolism. Studies have shown that CYP1A2 rs762551 AC/CC genotypes (associated with slower caffeine metabolism) are linked to increased risks of albuminuria, hyperfiltration, and hypertension with high coffee intake [43], supporting a genetic predisposition. These findings align with research demonstrating that CYP1A2 polymorphism impacts athletic performance [44,45], suggesting a broader influence on individual responses to caffeine. The interplay between climate, genetics, and individual factors likely shapes the complex response to caffeine, highlighting the need for personalized recommendations.

This narrative review aims to critically examine the role of caffeine in brain health among older adults, with a focus on its pharmacological mechanisms, cognitive and motor effects, potential neuroprotective properties in neurodegenerative diseases, and the often-overlooked issue of dependence. By integrating findings across diverse neurological domains, this review seeks to inform clinical awareness and public health strategies for safe and personalized caffeine consumption in later life.

## 2. Review Results

This narrative review presents an integrated synthesis of current evidence regarding the effects of caffeine on brain health in older adults. The findings are organized into thematic sections to reflect the most clinically and biologically relevant domains.

First, the pharmacokinetic and pharmacodynamic characteristics of caffeine are reviewed, with particular attention to age-related changes that influence its absorption, metabolism, and action on adenosine, dopamine, and glutamate systems. These mechanisms underpin both the stimulant effects of caffeine and its potential to induce tolerance and dependence.

The review then explores the neurobiological basis of caffeine dependence, including receptor adaptations and withdrawal syndromes, which remain under-recognized in elderly populations. Following this, the role of caffeine in various neurological and neurodegenerative conditions is assessed. In movement disorders, such as Parkinson’s disease and essential tremor, as well as in conditions like multiple sclerosis and Tourette’s syndrome, caffeine shows a spectrum of effects ranging from potentially protective to symptom-aggravating depending on dose, disease type, and individual sensitivity.

Attention is also given to caffeine’s influence on cognitive domains, including memory, attention, and executive function, as well as its debated role in age-related cognitive decline. Finally, the review evaluates emerging evidence of caffeine’s impact on major neurodegenerative diseases, including Alzheimer’s disease, vascular dementia, frontotemporal dementia, and Lewy body dementia.

Together, these results highlight a complex and sometimes contradictory profile of caffeine in later life, necessitating a personalized and cautious approach to its use.

### 2.1. Effects of Caffeine on the Brain: Pharmacokinetics and Pharmacodynamics

Caffeine, a psychoactive compound classified within the methylxanthine group, is widely consumed for its stimulant properties. Its pharmacological activity is intrinsically linked to its trajectory within the body, from absorption to elimination. The absorption of caffeine occurs rapidly, primarily through the gastrointestinal tract, with plasma concentrations reaching their peak approximately 30 to 60 min post-ingestion. Once in circulation, caffeine rapidly disseminates across tissues, including the central nervous system, due to its ability to cross the blood–brain barrier. The hepatic metabolism of caffeine plays a central role, principally mediated by the enzyme CYP1A2, a member of the cytochrome P450 system. This process generates active metabolites, including paraxanthine, theobromine, and theophylline, which contribute to the overall psychoactive effects. Renal excretion is the main pathway for the elimination of caffeine and its metabolites, with the half-life varying significantly among individuals due to factors like age, hepatic and renal function, smoking, and concurrent medication use [46,47,48,49]. In older adults, significant pharmacokinetic changes occur that warrant special attention. Hepatic metabolism, primarily through CYP1A2, declines with age, leading to slower clearance of caffeine [50,51,52,53]. Studies have shown that the half-life of caffeine can increase from 3–5 h in young adults to 6–10 h or more in older adults [19,54]. Reduced renal function further impairs the excretion of caffeine metabolites, prolonging their presence in the body [3,4,11,55]. Consequently, the same dose of caffeine can result in higher plasma concentrations and a greater risk of adverse effects in older adults.

As previously said, it is essential to underscore that individual response to caffeine is highly subjective and can be influenced by genetic predispositions, such as variants of the CYP1A2 gene, as well as physiological states, including pregnancy and aging [50,55,56,57]. In particular, aging-related changes can prolong caffeine’s half-life and heighten susceptibility to adverse effects, underscoring the importance of personalized consumption guidelines [46]. Therefore, healthcare providers should consider these age-related pharmacokinetic changes when recommending caffeine intake to older adults, carefully assessing potential interactions with medications and existing health conditions [2,58].

Caffeine exerts its primary pharmacodynamic effects mainly through antagonism of adenosine receptors in the central nervous system. Adenosine, an inhibitory neurotransmitter, accumulates during wakefulness, promoting relaxation and preparing the brain for sleep. Structurally similar to adenosine, caffeine binds to these receptors without activating them, functioning as a competitive antagonist. This blockade reduces the inhibitory influence of adenosine, thereby increasing arousal, alertness, and cognitive performance. Caffeine exhibits high affinity particularly for the A_1_ and A2A adenosine receptor subtypes [59,60,61].

The antagonism of A_1_ receptors, predominantly located in the hippocampus, cerebral cortex, and basal ganglia, facilitates the release of excitatory neurotransmitters, such as dopamine, acetylcholine, and glutamate. These mechanisms help explain caffeine’s stimulant effects on cognitive functions, mood, and vigilance [62,63,64,65,66]. Meanwhile, blocking A2Areceptors, mainly expressed in cerebral vasculature and the striatum, contributes to caffeine’s vasoconstrictive properties and influences motor control. Moreover, inhibition of A2Areceptors in the striatum enhances dopamine release, underpinning caffeine’s rewarding properties and its potential for dependence [67,68,69,70,71,72,73].

Beyond direct receptor interaction, caffeine modulates dopaminergic and cholinergic systems by increasing dopamine release and inhibiting its reuptake at the synaptic level, largely via antagonism of A2Areceptors [74,75,76]. The resultant elevation of dopaminergic activity in the striatum, a key brain area involved in motivation, reward, and executive function, contributes to enhanced mood, motivation, and cognitive performance [77,78]. However, this same mechanism also underpins the development of tolerance and dependence.

Additionally, caffeine influences the cholinergic system by promoting the release of acetylcholine in several brain regions, including the prefrontal cortex and the hippocampus. This effect is mainly mediated by the inhibition of A_1_ adenosine receptors and plays a significant role in facilitating cognitive processes, such as attention, learning, and memory [11,49].

### 2.2. Neurobiological Mechanisms of Caffeine Dependence: From Neurotransmitter Systems to Motor and Cognitive Circuits

Chronic caffeine consumption induces neuroadaptive changes that lead to tolerance and dependence. The brain, continually exposed to caffeine’s antagonistic effects on adenosine receptors, activates compensatory mechanisms to preserve homeostasis. One such mechanism is receptor down-regulation, characterized by a reduction in the number of adenosine A_1_ and A2Areceptors on neuronal cell surfaces. This decrease diminishes the brain’s sensitivity to caffeine’s effects, necessitating higher doses to achieve the same stimulating response. Such adaptation underpins the development of tolerance, whereby increasing amounts of caffeine are required to produce desired effects [79,80,81,82].

Abrupt cessation or significant reduction of caffeine intake following prolonged habitual use can precipitate withdrawal symptoms [83]. This occurs because the suppression of adenosine’s inhibitory action is no longer present, leading to a rebound effect. Withdrawal symptoms typically expressed are listed below.

- Headache: Adenosine acts as a vasodilator; caffeine’s vasoconstrictive effect contributes to headaches upon discontinuation due to sudden vasodilation.
- Fatigue and drowsiness: Without caffeine’s antagonism, adenosine binds to its receptors, promoting sleepiness and impaired concentration.
- Irritability, anxiety, and depressive symptoms: Caffeine elevates dopamine and other mood-related neurotransmitter levels; withdrawal can cause a chemical imbalance, resulting in symptoms of mood and anxiety disorders.
- Cognitive difficulties and psychomotor slowing: Caffeine enhances alertness and cognitive functions; withdrawal reduces these capabilities, leading to concentration deficits and slowed processing.
- Physical symptoms: Nausea, vomiting, and muscle pain are also reported [19,20,56,83,84,85,86].

Manifestations of caffeine dependence in the elderly may present unique challenges for clinical identification. While the core withdrawal symptoms remain consistent with younger populations, their presentation can be masked or misinterpreted due to the higher prevalence of comorbid conditions and age-related physiological changes [85,87,88,89]. For instance, fatigue, a common withdrawal symptom, can be easily attributed to the natural aging process or underlying medical conditions, such as anemia, endocrinopathies, or different metabolic disorders [90]. Similarly, headaches may be dismissed as tension headaches or attributed to medication side effects [91,92]. Irritability and anxiety may be misconstrued as symptoms of various neuropsychiatric disorders, including primary anxiety disorders, mood disorders, or early manifestations of a neurodegenerative condition [93,94].

Furthermore, the cognitive enhancing effects of caffeine can lead to a cycle of dependence, with older adults using caffeine to counteract age-related cognitive decline or fatigue, unknowingly perpetuating their dependence. This can make it difficult to distinguish between caffeine withdrawal symptoms and underlying cognitive impairment.

Clinical identification of caffeine dependence in the elderly requires a thorough assessment, including a detailed history of caffeine intake, a careful evaluation of other potential causes for their symptoms, and a high index of suspicion. Questionnaires designed to assess caffeine dependence, such as the Caffeine Use Disorder Questionnaire (CUDQ), may be useful in identifying problematic caffeine use [95], but their validity in older adults needs further investigation. Clinicians should also be aware of the potential for underreporting of caffeine consumption due to social stigma or lack of awareness [96,97].

Given the potential for misdiagnosis and the negative impact of caffeine dependence on health outcomes, clinicians should consider routine screening for caffeine use and dependence in older adults, particularly those presenting with unexplained fatigue, anxiety, or cognitive complaints. Strategies to manage caffeine dependence in older adults include gradual caffeine reduction, behavioral therapies, and management of comorbid conditions [21,98].

While caffeine does not directly bind to dopaminergic receptors, it indirectly activates the brain’s reward circuits, particularly the mesolimbic dopamine pathway. Originating in the ventral tegmental area (VTA) and projecting to the nucleus accumbens, amygdala, and prefrontal cortex, this system mediates feelings of pleasure, motivation, and associative learning [75]. Caffeine’s blockade of A2Aadenosine receptors in the striatum enhances dopamine release in the nucleus accumbens, producing pleasure and positive reinforcement that contribute to repeated consumption and dependence development.

The rewarding and cognitive-enhancing effects of caffeine are largely mediated through the increased release of excitatory neurotransmitters, including dopamine, acetylcholine, and glutamate, via the antagonism of adenosine receptors [99]. This mechanism explains caffeine’s efficacy in improving vigilance, attention, memory, and executive functions. However, this stimulatory effect can become self-perpetuating, as dependence develops as individuals seek to maintain optimal cognitive performance, fostering a vicious cycle. Tolerance to caffeine’s cognitive effects may drive increased intake, culminating in dependence and withdrawal symptoms, such as headache, fatigue, and irritability, upon attempts to reduce consumption [100].

Long-term habitual caffeine intake may lead to significant neurophysiological alterations, with potential negative consequences for cognitive function and motor control. Prolonged caffeine exposure induces down-regulation of adenosine receptors, resulting in modifications across several brain regions described below [65,77,101].

- Prefrontal cortex (PFC): The PFC is critical for executive functions, working memory, decision making, and attention. The PFC shows increased activity initially with caffeine due to elevated levels of dopamine and acetylcholine. Chronic use, however, can lead to dopamine receptor down-regulation, impairing cognitive efficiency, reducing cognitive flexibility, and increasing impulsivity.
- Hippocampus: Essential for episodic memory formation and learning, caffeine’s acute effects may enhance short-term memory; however, long-term intake can interfere with synaptic plasticity, which is necessary for long-term memory consolidation, potentially impairing learning and memory retention.
- Amygdala: Involved in emotion regulation, particularly fear and anxiety, caffeine may augment amygdala activity, heightening stress responses and anxiety, especially in predisposed individuals. Chronic use may contribute to hyperactivity of this region, exacerbating anxiety and irritability, notably during caffeine withdrawal.
- Striatum: Caffeine influences motor control primarily through antagonism of A2Areceptors in the striatum, a pivotal structure in voluntary movement regulation [102,103,104,105,106]. Persistent caffeine intake can cause desensitization of dopaminergic receptors in the striatum, diminishing its initial positive effects on motor coordination. Manifestations may include decreased movement precision, tremors, impaired coordination, and slowed reaction times.
- Cerebellum: This structure, integral for fine motor coordination, balance, and motor learning, may also be affected indirectly by caffeine-induced dopaminergic alterations, potentially contributing to deficits in balance and coordination. Although direct studies are limited, the neuroadaptive changes in dopaminergic pathways suggest that long-term caffeine consumption could subtly impair motor functions.

In summary, sustained caffeine intake can induce widespread alterations in neural activity across multiple brain regions, with potential adverse effects on cognitive and motor functions. It is crucial to recognize that individual sensitivity to caffeine, dosing patterns, and duration of use significantly modulate these neurophysiological responses, emphasizing the importance of personalized assessment and cautious consumption in vulnerable populations.

### 2.3. Caffeine and Movement Disorders

The impact of chronic caffeine use on movement disorders is a continually evolving area of research, characterized by ongoing debate. At present, there is no conclusive evidence to suggest that caffeine, by itself, predisposes individuals to specific movement disorders. However, given its complex interactions with the central nervous system, particularly with neurotransmitters, such as dopamine and adenosine, it remains plausible that caffeine may influence the manifestation or progression of certain motor pathologies.

Caffeine exerts its effects on various components of the motor pathway, including the following.

- Frontal cortical areas: Caffeine may increase cortical excitability, potentially affecting the planning and initiation of voluntary movements.
- Basal ganglia: The blockade of adenosine A2Areceptors within the striatum, part of the basal ganglia circuitry, influences dopamine release, which is crucial for motor control.
- Cerebellum: Although direct effects are less clearly established, caffeine might indirectly modulate cerebellar function, a region involved in coordination and motor learning.

There is currently no evidence to classify caffeine as a specific risk factor for movement disorders. On the contrary, some studies suggest it may have a protective role in certain conditions. Many epidemiological studies have found an inverse correlation between caffeine consumption and the risk of Parkinson’s disease (PD) [107]. This suggests that regular caffeine intake might reduce the risk of PD onset, possibly through adenosine receptor modulation. Caffeine acts as a non-selective antagonist of adenosine receptors, predominantly targeting the A2Asubtype, which is highly expressed in the basal ganglia, a brain region intimately involved in motor regulation and heavily affected in PD [108,109]. Supporting this, a recent PET imaging study showed that caffeine occupies A2Areceptors in PD patients, reinforcing the role of A2Areceptor antagonism in caffeine’s effects [110].

Specifically, the A2Areceptors, located on GABAergic neurons in the striatum, inhibit dopamine release when activated [111]. By blocking these receptors, caffeine reduces GABAergic inhibition and indirectly facilitates dopamine release, potentially restoring balance in the basal ganglia’s pathways disrupted in PD [112].

Caffeine’s inhibition of A2Areceptors may increase dopamine availability in the basal ganglia, potentially counteracting the characteristic dopaminergic deficit in PD [109,113]. Moreover, caffeine might enhance dopamine release by increasing tyrosine hydroxylase activity, the enzyme that limits dopamine synthesis [114,115]. Research has shown that caffeine can elevate dopamine levels in the striatum in animal PD models [107,116,117,118]. Recent studies indicate that individuals with early, untreated PD who consume high amounts of coffee exhibit reduced dopamine transporter binding in striatal regions, suggesting possible compensatory down-regulation due to chronic caffeine intake [119]. Additionally, a large Asian cohort study found that caffeine’s protective effect against PD was more pronounced in individuals without certain LRRK2 risk variants, indicating a gene–caffeine interaction [120]. A review identified several additional genes, including MAPT, SLC2A13, ApoE, NOS2A, GRIN2A, CYP1A2, and ADORA2A, that may interact with caffeine to modify PD risk, highlighting the genetic and environmental interplay in PD pathogenesis [121]. Furthering this evidence, a prospective analysis within the EPIC4PD cohort found a negative association between coffee consumption and PD risk. Prediagnostic plasma levels of caffeine and its primary metabolites (paraxanthine and theophylline) were also inversely associated with PD risk, supporting caffeine’s causal role in PD prevention [122]. Similarly, a prospective UK Biobank study found that higher intake of caffeinated coffee, particularly unsweetened, was linked to reduced PD risk, unlike sugar-sweetened or artificially sweetened varieties [123].

Besides adenosine antagonism and dopamine modulation, caffeine may also exert neuroprotective effects through mechanisms like reducing oxidative stress, inhibiting neuroinflammation, and modulating autophagy [107,118,124,125,126,127,128,129]. These combined mechanisms may help protect dopaminergic neurons from degeneration in PD.

Beyond its potential disease-modifying effects, caffeine might improve motor symptoms, such as rigidity and bradykinesia. A recent double-blind randomized controlled trial in Indonesia showed that caffeine adjunct therapy significantly improved motor function in PD patients, as measured by the UPDRS III score, compared to placebo, with only minor transient side effects [130]. Supporting caffeine’s potential in PD, preclinical research suggests that caffeine, especially combined with berberine, offers neuroprotection by reducing neuroinflammation and oxidative stress, critical factors in PD pathogenesis. This combination prevented motor deficits, dopamine depletion, and α-synuclein aggregation in a rotenone-induced rat model [114].

A clinical trial demonstrated that daily caffeine intake improved motor function in PD patients by modulating A2Areceptors and their regulatory role in the basal ganglia’s motor circuit [131]. A meta-analysis by Hong et al. highlighted that regular caffeine consumers have a significantly lower risk of developing PD.

However, recent findings indicate that lower salivary caffeine levels in PD patients are associated with greater disease severity, longer disease duration, and the presence of motor complications [132]. This suggests that the relationship between caffeine and PD progression is complex and possibly bidirectional; while caffeine might exert protective effects, more advanced stages of PD may lead to reduced caffeine intake due to medication interactions, side effects, or altered appetite.

Interestingly, among diagnosed patients, habitual caffeine consumption has been associated with slower disease progression, including a reduction in dyskinesia, motor fluctuations, and delayed initiation of levodopa therapy. These effects are hypothesized to result from A2Areceptor antagonism, which could mitigate neurotoxicity and neuroinflammation, key mechanisms involved in PD progression [133]. Nonetheless, it is important to remember that these associations do not establish causality definitively, and further randomized controlled trials are needed to confirm the therapeutic potential and optimal dosing of caffeine in PD.

The evidence regarding caffeine’s influence on motor symptoms remains mixed; while some studies report benefits, others show limited or no effects [131,133,134,135]. Larger, long-term investigations are essential to better understand whether caffeine can truly serve as an adjunct therapy for motor symptom management and disease progression in PD.

Beyond Parkinson’s, research also explores caffeine’s role in other neurodegenerative disorders. For example, multiple sclerosis (MS), an autoimmune condition of the central nervous system, has been associated with potential neuroprotective effects of caffeine. Observational studies, such as those by Hedström et al., suggest that high coffee consumption may be linked to a decreased risk of developing MS, possibly due to caffeine’s anti-inflammatory and neuroprotective properties [136]. Because adenosine blockade can reduce neuroinflammation and support neuronal health, caffeine might serve as a helpful adjunct in slowing disease progression [137]. Preliminary pilot studies have even reported improvements in balance and mobility in MS patients following caffeine intake; however, more controlled trials are necessary to draw definitive conclusions [138,139,140].

The relationship between caffeine and tremor disorders is still under investigation. Evidence suggests that caffeine’s stimulant effect could worsen tremor severity, especially in essential tremor, by increasing excitability in cerebellar and thalamic circuits involved in motor control [141,142]. However, some studies have shown no adverse effects or even slight improvements in tremor symptoms with caffeine, highlighting the variability in individual responses [143].

These contradictory findings may be due to differences in study design, caffeine dosages, sample sizes, or genetic factors influencing individual sensitivity. Notably, individuals with essential tremor or their first-degree relatives tend to consume less caffeine, possibly as a self-modification to manage symptoms, or due to increased sensitivity [144,145].

The relationship between caffeine and restless legs syndrome (RLS) is complex and not yet fully understood. RLS is a neurological disorder characterized by an irresistible urge to move the legs, often accompanied by uncomfortable sensations.

Some studies suggest that caffeine may exacerbate the symptoms of RLS, while others have found no significant correlation. Several mechanisms have been proposed to explain this potential interaction:

- Adenosine Antagonism: Caffeine acts as an antagonist at adenosine receptors, a neurotransmitter system that typically inhibits dopamine release and promotes relaxation and sleep. The adenosine theory of RLS posits that a deficiency of adenosine or dysfunction of its receptors within the central nervous system could contribute to the pathogenesis of RLS [146]. Because dopamine plays a crucial role in regulating movement and its imbalance is believed to be involved in RLS, the increased dopaminergic activity stimulated by caffeine might worsen symptoms in some individuals. By blocking adenosine receptors, caffeine could potentially exacerbate this imbalance, thereby intensifying symptoms.
- Sleep Disruption: As a well-known central nervous system stimulant, caffeine can interfere with sleep quality and duration. Given that RLS symptoms tend to intensify during evening and nocturnal hours, sleep deprivation caused by caffeine consumption may further aggravate the disorder [147].
- Iron Bioavailability: Iron deficiency is a recognized risk factor for RLS [148]. Although there is no conclusive evidence that caffeine directly impairs iron absorption, some studies suggest that high caffeine intake may interfere with intestinal iron uptake [149]. While the exact mechanism remains unclear, this interaction could contribute to worsening symptoms in predisposed individuals or those with latent iron deficiency [150].

A recent clinical practice guideline from the American Academy of Sleep Medicine recommends avoiding caffeine as part of first-line management strategies for RLS, especially in the evening [151].

The neural structures involved in RLS are not yet fully delineated, but current evidence indicates that dopaminergic pathways, the thalamus, and the hypothalamus are likely implicated. For example, one study identified an increased density of dopamine transporters in the striatum of RLS patients, suggesting altered dopaminergic regulation in this condition [152]. Although a direct causal relationship between caffeine and RLS has not been definitively established, existing evidence points to the possibility that caffeine may worsen symptoms in susceptible individuals or those already diagnosed with RLS.

Therefore, reducing or eliminating caffeine intake, particularly during evening hours, might be beneficial in managing RLS symptoms.

Dyskinesia is a movement disorder characterized by involuntary, often repetitive and uncontrollable movements. It can significantly impact patients’ quality of life, affecting their ability to perform daily activities. While various treatments are available for dyskinesia, their efficacy can vary, and many patients continue to experience debilitating symptoms. In recent years, increased interest has emerged in the potential therapeutic role of caffeine in certain types of dyskinesia, particularly those associated with mutations in the *ADCY5* gene. This gene encodes an enzyme called adenylate cyclase 5, which plays a crucial role in the production of cyclic AMP (cAMP), a key signaling molecule involved in neuronal function. Mutations in ADCY5 can lead to abnormal regulation of cAMP levels, which is believed to contribute to the development of dyskinesia [153].

Caffeine functions as an adenosine receptor antagonist. Adenosine is an inhibitory neurotransmitter within the central nervous system, exerting modulatory effects that promote neuronal relaxation and sleep. By blocking adenosine receptors, caffeine increases neuronal excitability and can influence downstream signaling pathways, including the production of cAMP [154]. Preliminary clinical studies have shown promising results regarding the use of caffeine for treating ADCY5-associated dyskinesia [154]. Patients with this condition have exhibited symptom improvements following caffeine consumption. Additionally, a retrospective study involving a larger cohort of ADCY5 mutation carriers indicated that caffeine was effective in reducing the frequency and duration of dyskinetic episodes in most cases [155].

It is important to note, however, that caffeine’s effects are not uniform across all forms of dyskinesia. For example, in PD, caffeine may sometimes exacerbate symptoms, highlighting the complexity of its influence depending on the underlying pathology [156].

While caffeine is not considered a direct cause of Tourette syndrome, some studies propose that it might increase tic severity in susceptible individuals [157]. This possible effect is thought to arise from caffeine’s capacity to elevate dopaminergic activity in the brain, as dopamine is a central neurotransmitter involved in motor control and believed to be dysregulated in Tourette syndrome. Müller-Vahl and colleagues documented a correlation between caffeine intake and tic worsening in a subset of Tourette patients. Nonetheless, it is essential to emphasize that sensitivity to caffeine varies considerably among individuals with Tourette syndrome; some may not notice any change or may even experience slight symptom relief [157].

Further research is necessary to clarify the relationship between caffeine and tics; however, in clinical practice, it may be advisable for individuals with Tourette syndrome to monitor their caffeine consumption and observe any effects on their symptoms. Diet and nutritional factors can influence symptom severity, and although more studies are needed to fully understand this connection, paying attention to dietary intake and individual responses can be an important aspect of managing symptoms. If tic severity appears to worsen after caffeine consumption, gradually reducing intake or eliminating caffeine altogether for a period may be beneficial, with subsequent evaluation to assess potential improvements [158].

### 2.4. Caffeine and Cognitive Domains

The long-term consumption of caffeine and its effects on the brain, particularly on neural structures and cognitive functions, have become topics of increasing scientific interest. While caffeine is generally regarded as safe and has been associated with certain short-term cognitive benefits, its long-term consequences, especially concerning brain architecture and functionality, remain subject to ongoing debate. Several observational studies have examined the relationship between habitual caffeine intake and structural brain changes, yet their findings are often conflicting. Some research suggests that high caffeine consumption may be associated with a reduction in gray matter volume in specific brain regions, such as the prefrontal cortex and the hippocampus [159]. These areas are essential for higher cognitive processes, including memory, attention, and executive functions. Importantly, it must be emphasized that correlation does not imply causation; these studies do not definitively demonstrate that caffeine directly causes such structural alterations, as other confounding factors, such as lifestyle, diet, and genetic predispositions, may also contribute.

Conversely, some evidence points to potential neuroprotective effects of caffeine. For instance, moderate caffeine intake has been linked to a decreased risk of developing neurodegenerative disorders, such as Alzheimer’s disease [160]. It is hypothesized that these benefits could be mediated through multiple mechanisms, including antioxidative properties, anti-inflammatory effects, and antagonism of adenosine receptors.

Regarding cognitive functions, long-term research presents a complex picture. While it is well-established that caffeine can acutely improve attention, vigilance, and cognitive performance, particularly in sleep-deprived states, the implications for long-term cognitive health are less conclusive. Some studies propose that habitual caffeine consumption might contribute to a slower rate of cognitive decline with aging, although other investigations have failed to find this association [65]. Functional magnetic resonance imaging (fMRI) studies have demonstrated that chronic caffeine consumers may exhibit altered resting-state brain activity compared to non-users. For example, reduced functional connectivity among different brain regions at rest has been observed in those with high caffeine intake. Caffeine also appears capable of modulating brain responses during demanding cognitive tasks; some research reports decreased activity in the prefrontal cortex, an area involved in executive function, among regular caffeine consumers at rest. However, during complex cognitive challenges, caffeine may increase activity in the same areas, possibly suggestive of compensatory mechanisms [161,162,163].

It is crucial to recognize that these observed alterations in brain activity from fMRI studies are not necessarily indicative of pathology or impairment. The brain is a highly plastic organ capable of adapting to various stimuli and habits, and changes induced by caffeine may reflect adaptive or compensatory responses rather than adverse effects.

### 2.5. Caffeine and Degenerative Diseases

#### 2.5.1. Caffeine and Alzheimer’s Disease

Alzheimer’s disease, with its inexorable progression, casts a dark shadow over the lives of millions, gradually eroding cognitive faculties and the most cherished memories. Despite relentless efforts by the scientific community, the etiological factors underlying this neurodegenerative disorder remain largely concealed within an enigmatic framework, and a definitive cure remains a distant goal. In recent years, renewed attention has been directed toward the potential influence of modifiable lifestyle factors, such as diet and the intake of certain substances, in modulating the risk of developing Alzheimer’s disease. Among these, caffeine has attracted considerable interest, fueling the faint hope of at least partially attenuating the devastating course of the disease.

Epidemiological studies have explored the association between caffeine consumption and Alzheimer’s risk, producing promising but still inconclusive results. Some observational research suggests that moderate caffeine intake, primarily via coffee, may be linked to a reduced risk of developing Alzheimer’s disease and age-related cognitive decline [164]. However, other studies have failed to confirm this association or have reported conflicting outcomes [165,166]. Despite this uncertainty, experimental evidence from in vitro and animal models points toward potential neuroprotective effects of caffeine in the context of Alzheimer’s pathology.

Adding to this complex picture, a recent review of the literature on caffeine and Alzheimer’s concluded that while clinical studies offer suggestive evidence of caffeine’s neuroprotective role against dementia and possibly AD, further research is necessary to confirm this link and to elucidate the specific mechanisms involved [167]. Moreover, another review highlighted the neuroprotective potential of Robusta coffee (Coffea canephora), a widely consumed variety, and its bioactive compounds, including caffeine and chlorogenic acids, in Alzheimer’s and other neurodegenerative conditions, suggesting that certain components of Robusta coffee may contribute to its neuroprotective effects [168].

Furthermore, a comprehensive review of caffeine’s mechanisms underscores its role as a non-selective adenosine receptor antagonist with antioxidant and anti-inflammatory properties. The review summarizes evidence from epidemiological and clinical studies indicating a reduced risk of Alzheimer’s disease, Parkinson’s disease, and dementia with caffeine intake [127]. In addition, low caffeine consumption has been associated with a higher likelihood of being amnestic among patients with mild cognitive impairment (MCI) or AD, and caffeine intake correlates with cerebrospinal fluid biomarkers in AD patients [169]. Supporting these findings, Wang et al. (2025) recently demonstrated that higher caffeine intake appears to be linked to better cognitive performance among older adults [170]. 

In contrast, a recent systematic review and meta-analysis of cohort studies found that although increased tea consumption was associated with a decreased risk of dementia and AD, the link between coffee intake and AD risk was not statistically significant. Nonetheless, the meta-analysis revealed a non-linear relationship, with moderate coffee consumption (one to three cups per day) showing a protective association [171].

Caffeine primarily functions as an antagonist of adenosine receptors in the brain. Adenosine, a neuromodulator involved in sleep regulation, neuronal excitability, and inflammatory responses, is found at elevated levels in the brains of Alzheimer’s patients, a condition believed to contribute to disease pathogenesis [137,172,173,174,175].

By blocking adenosine receptors, caffeine may exert anti-inflammatory effects, which are particularly relevant given that chronic inflammation is a key driver of Alzheimer’s progression. Furthermore, caffeine has demonstrated anti-inflammatory properties by reducing the production of pro-inflammatory cytokines within the brain. It may also inhibit the formation and accumulation of amyloid plaques, which are toxic aggregates of beta-amyloid peptides, considered a hallmark of Alzheimer’s pathology [176,177,178]. In vitro studies and animal experiments suggest that caffeine might interfere with the genesis of these plaques, potentially slowing neurodegeneration [179,180,181]. Additionally, caffeine’s acute cognitive benefits, such as improvements in attention, vigilance, and overall cognitive performance, are well-documented. Some research hypothesizes that these short-term effects could translate into long-term benefits, possibly decelerating age-associated cognitive decline [160,176].

Despite encouraging preliminary evidence, further research is essential to elucidate the precise role of caffeine in the prevention and treatment of Alzheimer’s disease. Well-designed randomized controlled trials are crucial to validate observational data, establish optimal dosing protocols, and determine appropriate treatment durations. Only through rigorous clinical investigation can the true potential of caffeine as a neuroprotective agent in Alzheimer’s disease be accurately assessed.

#### 2.5.2. Caffeine and Amyotrophic Lateral Sclerosis

Amyotrophic Lateral Sclerosis (ALS) is a progressive neurodegenerative disease characterized by the degeneration of motor neurons. Currently, there is no cure for ALS, and its underlying causes remain largely unknown. Given the disease’s complexity and the lack of effective treatments, research has increasingly focused on identifying modifiable risk factors and exploring potential therapeutic interventions. In this context, the role of caffeine has garnered some scientific interest; however, the evidence remains limited and inconclusive.

Several epidemiological studies have investigated the possible association between caffeine consumption and ALS risk, often yielding conflicting results. Two large-scale studies, one conducted by Fondell et al. involving over one million participants and an aggregated analysis of eight prospective cohort studies by Petimar et al., found no significant correlation between the intake of caffeine, coffee, or tea and the risk of developing ALS or related mortality [182,183]. Conversely, a separate study published in 2011 suggested that coffee might have a potential protective effect against ALS [184].

A limited number of studies have examined the impact of caffeine on the disease’s progression, but the current evidence remains too weak to draw definitive conclusions. A recent multicenter cross-sectional study evaluated the influence of coffee and tea consumption on ALS progression using the ALS Functional Rating Scale—Revised (ALSFRS-R), a validated tool for monitoring disease progression over time. The findings did not reveal a significant association between caffeine intake and the rate of disease progression [185].

In summary, while the potential neuroprotective effects of caffeine in ALS remain an intriguing area of investigation, the current scientific evidence is insufficient to establish a clear relationship.

#### 2.5.3. Vascular Dementia and Chronic Caffeine Use

Unlike Alzheimer’s disease, vascular dementia (VaD) is caused by a series of vascular pathologies that impair cerebral blood flow, leading to brain damage and cognitive decline. Given caffeine’s effects on the cardiovascular system and the vascular nature of VaD, it is essential to examine the potential interactions between these two factors.

Effects of caffeine on the vascular system include the following [186].

(a) Vasoconstriction: Caffeine is a well-known vasoconstrictor, meaning it can narrow blood vessels and temporarily elevate blood pressure. This effect is primarily mediated through antagonism of adenosine receptors–neurotransmitter receptors that normally promote vasodilation.
(b) Blood pressure elevation: Several studies have demonstrated that caffeine intake can induce transient increases in both systolic and diastolic blood pressure.
(c) Effects on endothelial function: The endothelium, the inner lining of blood vessels, plays a crucial role in regulating blood flow and arterial pressure. Some research suggests that caffeine may exert both beneficial and detrimental effects on endothelial function, depending on the dosage and duration of exposure.

On the other hand, observational studies indicate that moderate caffeine consumption might be associated with a reduced risk of developing dementia, including VaD [8,187,188,189]. This protective effect could be attributed to caffeine’s antioxidant and anti-inflammatory properties, combined with its capacity to enhance cerebral blood flow, acting as a fragile barrier against vascular insults [190,191,192,193]. Nonetheless, the picture remains uncertain. The vasoconstrictive action of caffeine, with its potential negative impact on endothelial health in susceptible individuals, could paradoxically increase the risk of cerebrovascular events, such as ischemic strokes, thereby elevating the likelihood of VaD.

As is often the case in the complex realm of health, the dose of caffeine and the individual’s response play pivotal roles, shaping a fine line between benefit and harm. While moderate consumption may be tolerated or even confer benefits, excessive intake could prove harmful, especially for individuals with a history of cardiovascular disease or hypertension, who need to navigate this territory with particular caution.

Currently, the scientific evidence regarding the relationship between chronic caffeine consumption and vascular dementia remains limited and inconclusive. In the meantime, a cautious approach is advisable; moderation in caffeine intake should be maintained, particularly among those at increased cardiovascular risk or with a familial history of VaD. Continued research is necessary to clarify these interactions and to guide evidence-based recommendations for caffeine consumption in relation to vascular cognitive decline.

#### 2.5.4. Caffeine and Frontotemporal Dementia

Frontotemporal dementia (FTD) presents a significant challenge within the landscape of neurodegenerative disorders, with its underlying mechanisms still largely elusive. While ongoing research continues to explore the complex pathophysiological processes involved, increasing emphasis is being placed on modifiable risk factors, such as lifestyle choices, which may influence disease onset and progression. In this context, the role of caffeine intake warrants careful consideration.

Caffeine primarily exerts its effects in the brain by antagonizing adenosine receptors, which are involved in neuroprotection and the regulation of neurotransmission. By blocking these receptors, caffeine increases the availability of other neurotransmitters, notably dopamine, noradrenaline, and, importantly, glutamate. Although most studies have concentrated on AD, these mechanisms offer intriguing insights relevant to FTD, as well. Specifically, the influence of caffeine on the glutamatergic system has garnered interest, albeit with necessary caution due to the paucity of research specifically targeting FTD.

Excessive activation of glutamate receptors, known as excitotoxicity, is a well-recognized mechanism of neuronal damage in various neurodegenerative conditions, including FTD. Given that caffeine can increase glutamate availability, it is plausible to hypothesize that high, chronic consumption, particularly in genetically predisposed individuals with dysregulation of the glutamatergic system, might exacerbate excitotoxic damage. This could contribute to the initial development and acceleration of neurodegeneration associated with FTD [8,169,194,195].

This hypothesis, suggesting a dose-dependent duality of caffeine’s effects, indicates that moderate intake might have no adverse consequences and could potentially confer neuroprotective benefits owing to its antioxidative and anti-inflammatory properties. Conversely, excessive and sustained consumption, especially in genetically susceptible individuals, could be detrimental, possibly triggering or hastening neurodegenerative processes. Although primarily based on indirect evidence, this proposition warrants further investigation through targeted studies examining the relationship between caffeine consumption, genetic predispositions, and the risk of FTD. Such research could ultimately inform dietary recommendations aimed at reducing the burden of these complex neurodegenerative conditions.

#### 2.5.5. Caffeine and Lewy Body Dementia

Lewy body dementia (LBD) is the second most common form of dementia after AD. It is characterized by the presence of abnormal protein aggregates, known as Lewy bodies, within neurons of the brain. The clinical presentation of LBD can include cognitive and executive dysfunction, behavioral changes, visual hallucinations, motor symptoms similar to PD, and sleep disturbances.

The association between caffeine consumption and the risk of dementia, including LBD, has been extensively investigated through epidemiological studies, yielding sometimes conflicting results.

Cornelis et al. examined the relationship between caffeine intake and dementia, with particular focus on the role of Lewy bodies. Their study analyzed data from two large cohorts: the Rush Memory and Aging Project (RMAP) and the UK Biobank (UKB) [196]. The researchers investigated associations among caffeine consumption, dementia incidence, cognitive decline, and Lewy body pathology. In the RMAP cohort, caffeine intake exceeding 100 mg per day was associated with a reduced likelihood of having Lewy bodies upon autopsy, although it was not linked to a decreased risk of clinical dementia or cognitive deterioration. Conversely, in the UKB cohort, moderate caffeine intake was associated with a lower risk of all-cause dementia and AD.

The authors propose that caffeine might confer neuroprotection by inhibiting Lewy body pathology, and they suggest that discrepancies in the epidemiological literature regarding caffeine and clinically diagnosed dementia could partly be due to differences in underlying causes across studied cohorts. Although the study provides compelling insights into the potential role of caffeine in modulating Lewy body pathology, further research is necessary to validate these findings and elucidate the underlying mechanisms involved in this relationship.

Given the widespread consumption of caffeine among older individuals and its complex interaction with brain function, a balanced overview of both neuroprotective effects and potential risks is essential. 

See Table 1 for a summary of key neurological effects associated with caffeine use in the elderly population.

## 3. Discussion

This narrative review highlights the multifaceted relationship between caffeine consumption and neurological health in the elderly, a subject that remains both clinically relevant and scientifically debated. The findings suggest that moderate caffeine intake may offer cognitive benefits, including enhanced attention, verbal fluency, and short-term memory performance [7,8,9]. These effects are attributed primarily to caffeine’s action as an adenosine receptor antagonist, resulting in increased arousal and improved cognitive efficiency. However, such benefits must be weighed against the physiological changes associated with aging, which can modify the pharmacokinetics and pharmacodynamics of caffeine.

In older adults, the reduced rate of hepatic metabolism and renal clearance leads to prolonged caffeine half-life and heightened systemic exposure [3]. This contributes to increased sensitivity to its effects, raising the risk of side effects, such as insomnia, irritability, tremor, anxiety, and cardiac arrhythmias [13,14,15]. Additionally, older individuals are more likely to experience dehydration, osteoporosis, and polypharmacy, all of which can interact with caffeine in clinically significant ways [16,17,18]. These considerations underscore the importance of personalized approaches when evaluating caffeine intake in the elderly population (Table 2).

While acute cognitive benefits are well-documented, long-term effects are less clear. Some studies suggest that habitual caffeine consumption may slow the trajectory of age-related cognitive decline and lower the risk of Alzheimer’s disease, Parkinson’s disease, and other neurodegenerative disorders [107,160,176]. In Parkinson’s disease specifically, caffeine appears to exert a protective role by enhancing dopaminergic transmission through A2Areceptor antagonism [109,131]. Conversely, in essential tremor and restless legs syndrome, caffeine may exacerbate symptoms due to increased excitability of motor pathways and interference with sleep [142,146]. These divergent effects suggest that the role of caffeine in neurological disorders is highly context dependent and must be assessed on a case-by-case basis (Table 3).

Caffeine dependence remains an under-recognized issue in older populations. Tolerance, withdrawal symptoms, such as headache, fatigue, and depressed mood, and continued use despite harm meet the diagnostic criteria for a substance use disorder in some cases [19,20,21]. Although the prevalence of clinically significant caffeine dependence in older adults is not well-documented, age-related physiological changes may enhance vulnerability. The drive to preserve cognitive function or counteract fatigue may reinforce habitual use, increasing the likelihood of dependence over time [30,32] (Table 4).

From a broader neurobiological perspective, chronic caffeine exposure can lead to down-regulation of adenosine receptors and altered activity in several brain regions, including the prefrontal cortex, hippocampus, and striatum [65,77,101]. These changes may impair executive function, memory consolidation, and emotional regulation, particularly in vulnerable individuals. The modulation of dopaminergic, cholinergic, and glutamatergic systems by caffeine is central to both its cognitive-enhancing and potentially detrimental effects [76,99].

With regard to dementia, emerging evidence points to a possible protective association between moderate caffeine intake and a reduced risk of Alzheimer’s disease and Lewy body dementia, potentially due to anti-inflammatory and antioxidant effects [178,179,196]. However, inconsistencies across studies and methodological heterogeneity hinder definitive conclusions. In vascular dementia, caffeine’s vasoconstrictive effects may present a double-edged sword, with potential to either impair or support cerebral perfusion depending on the clinical context [190,191]. The limited but intriguing findings on caffeine in frontotemporal dementia also suggest that glutamatergic dysregulation may exacerbate neurodegeneration in susceptible individuals [169].

Another dimension is the variability in individual responses, shaped by genetic polymorphisms (e.g., CYP1A2 variants), concurrent medication use, and cultural factors influencing patterns of caffeine intake [11,37,50]. This diversity necessitates a move away from universal recommendations toward personalized guidance in clinical practice. The form of caffeine consumption—coffee, tea, energy drinks, or pharmaceuticals—also affects absorption rates, co-ingested compounds, and overall impact on the central nervous system [2,36].

In summary, while caffeine remains one of the most widely used psychoactive substances worldwide, its role in promoting brain health in older adults is complex and context-sensitive. The challenge for clinicians and public health professionals lies in developing balanced, individualized advice that recognizes both the benefits and risks. There is a pressing need for longitudinal studies and randomized controlled trials that focus specifically on older populations to clarify the long-term effects of caffeine on cognitive decline, neurological disease progression, and dependence. Public health strategies should incorporate education about safe caffeine use, especially for those with sleep disorders, cardiovascular risk, or neurodegenerative conditions. By integrating epidemiological data, clinical observations, and neurobiological insights, more nuanced and age-appropriate recommendations can be developed to support cognitive and neurological health in aging societies (Table 5).

### Limitations

This review has several limitations that should be considered when interpreting its findings.

Firstly, the definition of “elderly people” varies across studies. While we have chosen to define “elderly people” as individuals aged 65 years or older for the sake of clarity, it is important to acknowledge that many studies included in this review employ different age-based inclusion criteria, ranging from 60 years and older to other age ranges. This variability may limit the comparability of results across studies and introduce heterogeneity in the analyzed populations, potentially affecting the generalizability of the findings. Secondly, the majority of studies included in this review are observational in nature. Observational studies can only demonstrate associations, not causation. This means that we cannot definitively conclude that caffeine directly causes the observed effects on cognitive function, neurodegeneration, or addiction. Furthermore, observational studies are susceptible to various biases, such as selection bias (where participants who choose to consume caffeine may differ systematically from those who do not, potentially skewing results), information bias (due to reliance on self-reported caffeine intake, which may be inaccurate), and confounding factors (where other factors, such as lifestyle, diet, or genetics, may influence both caffeine consumption and the outcomes of interest, making it difficult to isolate the specific effect of caffeine).

Thirdly, there is a paucity of studies specifically analyzing the effects of caffeine in individuals over 80 years old, despite the fact that this age group is particularly vulnerable to the adverse effects of caffeine due to age-related physiological changes (e.g., reduced hepatic and renal function), increased comorbidity, and polypharmacy. Therefore, findings from studies on younger elderly populations may not be directly generalizable to this vulnerable subgroup, and caution should be exercised when extrapolating results.

Fourthly, the varying sources of caffeine (coffee, tea, energy drinks, medications) across studies pose a challenge. These sources differ not only in caffeine concentration but also in the presence of other bioactive compounds (e.g., antioxidants, polyphenols) that may interact with caffeine or exert independent effects on the brain, potentially confounding the results and making it difficult to isolate the specific effects of caffeine.

Fifthly, the contradictory findings regarding the effects of caffeine on certain conditions, such as essential tremor, highlight the complexity of this topic. As discussed earlier, these contradictions may stem from differences in study design (e.g., cross-sectional vs. longitudinal), sample size, caffeine dosage, administration methods, and individual variability in caffeine sensitivity. It is also important to note that individuals with essential tremor, or their first-degree relatives, may modify their caffeine consumption in response to tremor symptoms, potentially influencing the observed associations.

Finally, many studies rely on self-reported caffeine intake, which is subject to recall bias and social desirability bias, leading to underreporting or inaccurate estimates of caffeine consumption. Objective measures of caffeine consumption, such as plasma caffeine levels or urinary caffeine metabolites, are rarely used, limiting the accuracy of exposure assessment and potentially affecting the validity of study findings.

## 4. Conclusions

Caffeine consumption in older adults presents a nuanced balance between potential cognitive benefits and physiological vulnerabilities. While moderate intake may support attention and delay neurodegenerative progression in selected conditions, age-related changes in metabolism and sensitivity call for caution. The risks of sleep disturbances, cardiovascular effects, and interaction with polypharmacy cannot be overlooked.

Equally relevant is the often overlooked potential for dependence. Chronic use may lead to tolerance and withdrawal symptoms that interfere with daily functioning, sometimes misinterpreted as primary geriatric complaints.

Given its widespread use and complex effects, caffeine should not be considered universally benign in later life. Clinical practice and public health efforts must promote personalized guidance, considering comorbidities, lifestyle, and cognitive status. While individual needs vary, general recommendations suggest that caffeine intake should not exceed 400 mg per day for most healthy adults seeking cognitive enhancement [54,65]. However, this level may need to be adjusted based on individual brain health factors. For example, individuals with existing sleep disorders or anxiety should limit or avoid caffeine to minimize the risk of exacerbating these conditions and negatively impacting cognitive function.

It is crucial to recognize that the misuse or excessive consumption of caffeine, particularly in combination with other substances, can worsen underlying psychiatric conditions like anxiety, sleep disorders, and mood disturbances, potentially fueling neuroinflammatory processes. Moreover, withdrawal from caffeine can also trigger neuroinflammation, further complicating the clinical picture. In individuals at risk of neurodegenerative diseases, the potential benefits of caffeine should be carefully weighed against the risks of dependence and other adverse effects.

Ultimately, the optimal caffeine intake for cognitive enhancement and neuroprotection should be determined on an individual basis in consultation with a healthcare professional, considering their specific cognitive profile, risk factors, and susceptibility to dependence. The presence of neuroinflammatory conditions, which may be modulated by caffeine’s anti-inflammatory properties [197], should also be considered.

Future studies, particularly longitudinal ones, are essential to clarify the long-term impact of caffeine on brain health and inform evidence-based recommendations for the aging population.

## Figures and Tables

**Table 1:** Caffeine use in the elderly: neuroprotective potential and neurological risks.

Neuroprotective Potential	Neurological Risks
Reduced risk of Parkinson’s disease [107,133]	Increased anxiety and sleep disturbances [14,15]
Possible delay in onset of Alzheimer’s disease [160,176]	Potential worsening of essential tremor and restless legs syndrome [142,146]
Modulation of adenosine receptors contributing to cognitive enhancement [174,175]	Possible reduction in gray matter volume with high intake [21,159]
Anti-inflammatory and antioxidant effects potentially beneficial in dementia [137,178]	Risk of tolerance and withdrawal symptoms, such as headache, irritability, and fatigue [20,21]
Improved attention and vigilance [65]	Potential glutamatergic excitotoxicity in frontotemporal dementia [169,195]

**Table 2:** Caffeine metabolism in the elderly: pharmacokinetic and pharmacodynamic changes.

Aspect	Young Adults	Older Adults	References
Caffeine half-life	3–5 h	6–10+ h	[3]
Enzyme activity (CYP1A2)	Normal	Reduced	[47]
Renal clearance	Efficient	Often reduced	[17]
Central sensitivity	Moderate	Increased	[10]

**Table 3:** Caffeine and neurological disorders: summary of evidence.

Condition	Effect	Direction	References
Parkinson’s Disease	Protective	↓ Risk	[107,133]
Lewy Body Dementia	Possibly protective	↓ Pathology	[196]
Alzheimer’s Disease	Inconclusive	?	[160]
Restless Legs Syndrome	Exacerbating	↑ Symptoms	[146]
Frontotemporal Dementia	Potentially harmful	↑ Excitotoxicity	[195]

**Table 4:** Caffeine and neurological disorders: summary of evidence.

Symptom	Description	References
Headache	Due to vasodilation rebound; misattributed to migraine/hypertension	[20]
Fatigue	Adenosine rebound activity; can mimic chronic fatigue	[21]
Irritability	Dopaminergic imbalance; may resemble depression	[19]
Sleepiness	Unopposed adenosine; often confused with aging effects	[14]

**Table 5:** Caffeine and neurological disorders: summary of evidence.

Recommendation	Rationale	Risk Profile
Limit intake to <300 mg/day	To reduce insomnia, anxiety, withdrawal	All elderly
Avoid caffeine in the evening	To improve sleep quality	Insomniacs, RLS patients
Monitor for signs of dependence	Headaches, fatigue, tolerance	Chronic consumers
Consider genetics and comorbidities	CYP1A2 polymorphisms, polypharmacy	Frail elderly

## Data Availability

No new data were created or analyzed in this study. Data sharing is not applicable to this article.
